# Supplementary material for: The Stress Response of the Holothurian Central Nervous System: A Transcriptomic Analysis
Source: Int J Mol Sci. 2022 Nov 2;23(21):13393. doi: 10.3390/ijms232113393 (PMC9657328; doi:10.3390/ijms232113393)
Supplement: Supplementary file 1 [file ijms-23-13393-s001.zip › ijms-1972311-supplementary.pdf]

Supplementary Materials

A

```
TRINITY_DN33848    1 -QIFVKTLPGKTITLEVEPSDTIENVKSNIQDKEGIPPDQQRLIFSGKQLEDGRTLYDYN
AAH25894.1         1 MQIFVKTLTGKTITLEVEPSDTIENVKAKIQDKEGIPPDQQRLIFAGKQLEDGRTLSDYN
NP_001284937.1     1 MQIFVKTLTGKTITLEVEPSDTIENVKAKIQDKEGIPPDQQRLIFAGKQLEDGRTLSDYN
PIK46166.1         1 MQIFVKTLTGKTITLEVEPSDTIENVKSKIQDKEGIPPDQQRLIFAGKQLEDGRTLSDYN

TRINITY_DN33848   60 IQKEATLHLVVRLRGGMQIFVKTLPGKTITLEVEPSDTIENVKSNIQDKEGIPPDQQRLI
AAH25894.1        61 IQKESTLHLVLRLRGGMQIFVKTLTGKTITLEVEPSDTIENVKAKIQDKEGIPPDQQRLI
NP_001284937.1    61 IQKESTLHLVLRLRGGMQIFVKTLTGKTITLEVEPSDTIENVKAKIQDKEGIPPDQQRLI
PIK46166.1        61 IQKESTLHLVLRLRGGMQIFVKTLTGKTITLEVEPSDTIENVKSKIQDKEGIPPDQQRLI

TRINITY_DN33848  120 FSGKQLEDGRTLYDYNIQKEATLHLVVRLRGGMQIFVKTLTGKTITLEVEPSDTIENVKT
AAH25894.1       121 FAGKQLEDGRTLSDYNIQKESTLHLVLRLRGGMQIFVKTLTGKTITLEVEPSDTIENVKA
NP_001284937.1   121 FAGKQLEDGRTLSDYNIQKESTLHLVLRLRGGMQIFVKTLTGKTITLEVEPSDTIENVKA
PIK46166.1       121 FAGKQLEDGRTLSDYNIQKESTLHLVLRLRGGMQIFVKTLTGKTITLEVEPSDTIENVKS

TRINITY_DN33848  180 KNQDKQGIP---------------------------------------------------
AAH25894.1       181 KIQDKEGIP---------------------------------------------------
NP_001284937.1   181 KIQDKEGIPPDQQRL-IFAGKQL------------EDGRTLSDYNIQKESTLHLVLRLRG
PIK46166.1       181 KIQDKEGIPQISSVLSLLVSSSRTEGRYPTTTSRKSPHSILSFVSVVKESTLHLVLRLRG

TRINITY_DN33848  189 --------------------------------------P---------------YQQRKI
AAH25894.1       190 --------------------------------------P---------------DQQRLI
NP_001284937.1   228 GMQIFVKTLTGKTITLEVEPSDTIENVKAKIQDKEGIPP---------------DQQRLI
PIK46166.1       241 GMQIFVKTLTGKTITLEVEPSDTIENVKSKIQDKEGIPPDQQRLIFAGQGRYSPDQQRLI

TRINITY_DN33848  196 FAGFPLDDGRTLSDYNIQKESTLHLVLRLRGGMQIFDKTLTGKTITLEVEPSDTIENVKT
AAH25894.1       197 FAGKQLEDGRTLSDYNIQKESTLHLVLRLRGGMQIFVKTLTGKTITLEVEPSDTIENVKA
NP_001284937.1   273 FAGKQLEDGRTLSDYNIQKESTLHLVLRLRGGMQIFVKTLTGKTITLEVEPSDTIENVKA
PIK46166.1       301 FAGKQLEDGRTLSDYNIQKESTLHLVLRLRGGMQIFVKTLTGKTITLEVEPSDTIENVKS

TRINITY_DN33848  256 K-----------------------------------------------------------
AAH25894.1       257 K-----------------------------------------------------------
NP_001284937.1   333 KIQDKEGIPPDQQRLIFAGKQLEDGRTLSDYNIQKESTLHLVLRLRGGMQIFVKTLTGKT
PIK46166.1       361 KIQDKEGIPPDQQRLIFADL--------------------------------CQNSDWQD

TRINITY_DN33848  257 -----------------NQDKQGIPPYQQRKIFAGFPLDDGRTLSDYNIQKESTLHLVLR
AAH25894.1       258 -----------------IQDKEGIPPDQQRLIFAGKQLEDGRTLSDYNIQKESTLHLVLR
NP_001284937.1   393 ITLEVEPSDTIENVKAKIQDKEGIPPDQQRLIFAGKQLEDGRTLSDYNIQKESTLHLVLR
PIK46166.1       389 HTLEVEPSDTIENVKSKIQDKEGIPPDQQRLIFAGKQLEDGRTLSDYNIQKESTLHLVLR

TRINITY_DN33848  300 LRGGMQIFDKTLTGKT--------------------------------------------
AAH25894.1       301 LRGGMQIFVKTLTGKTITLDVEPSVTTKKVKQEDRRTFLTTVSKKSPPCACSW-------
NP_001284937.1   453 LRGGMQIFVKTLTGKTITLEVEPSDTIENVKAKIQ------DKEGIPPDQQRLIFAGKQL
PIK46166.1       449 LRGGMQIFVKTLTGKTITLEVEPSDTIENVNPKSG------QGRYSPDHSVSSLLVNN--

TRINITY_DN33848      ----------------------------
AAH25894.1       354 ---------------------------V
NP_001284937.1   507 EDGRTLSDYNIQKESTLHLVLRLRGGAI
PIK46166.1           ----------------------------
```

B

```
TRINITY_DN43805     1 ADGRSLYDFNLPKESTLHVVLRLRGCMQIFVNTLTGKTITLEVETSDTIENVKSKIQDKE
AAA28997.1          1 MQIFVKTLTGKTITLEVEPSDTIENVKAKIQDKEGIPPDQQRLIFAGKQLEDGRTLSDYN
AAH08661.1          1 MQIFVKTLTGKTITLEVEPSDTIENVKAKIQDKEGIPPDQQRLIFAGKQLEDGRTLSDYN
PIK45544.1          1 MQIFVKTLTGKTITLEVEPSDTIENVKSKIQDKEGIPPDQQRLIFAGKQLEDGRTLSDYN

TRINITY_DN43805    61 GIPPDQQRLIIFPGKQLEDGRTQYQTNIHHNETTRQ-PNRYTE-----------------
AAA28997.1         61 IQKESTLHLVLRLRGGMQIFVKTLTGKTITLEVEPSDTIENVKAKIQDKEGIPPDQQRLI
AAH08661.1         61 IQKESTLHLVLRLRGGMQIFVKTLTGKTITLEVEPSDTIENVKAKIQDKEGIPPDQQRLI
PIK45544.1         61 IQKESTLHLVLRLRGGMQIFVKTLTGKTITLEVEPSDTIENVKSKIQDKEGIPPDQQRLI

TRINITY_DN43805       ------------------------------------------------------------
AAA28997.1        121 FAGKQLEDGRTLSDYNIQKESTLHLVLRLRGGMQIFVKTLTGKTITLEVEPSDTIENVKA
AAH08661.1        121 FAGKQLEDGRTLSDYNIQKESTLHLVLRLRGGMQIFVKTLTGKTITLDVEPSVTTKKVKQ
PIK45544.1        121 FAGKQLEDGRTLSDYNIQKESTLHLVLRLRGGMQIFVKTLTGKTITLEVEPSDTIENVKS

TRINITY_DN43805       ---------------------------------------------------
AAA28997.1        181 KIQDKEGIPPDQQRLIFAGKQLEDGRTLSDYNIQKESTLHLVLRLRGGIQA
AAH08661.1        181 EDRRTFLTTVSKKSPPCACSWV-----------------------------
PIK45544.1        181 KIQDKEGIPQTSSVSSSLVNN------------------------------
```

C

```
TRINITY_DN16432     1 ----VKTLTGKTITLDVEPSDTIENVKSKIQDKEGIPPDQQSLIFAGKQLEDGRTLSDYN
BAB22630.1          1 MQIFVKTLTGKTITLEVEPSDTIENVKAKIQDKEGIPPDQQRLIFAGKQLEDGRTLSDYN
AAA28997.1          1 MQIFVKTLTGKTITLEVEPSDTIENVKAKIQDKEGIPPDQQRLIFAGKQLEDGRTLSDYN
PIK45544.1          1 MQIFVKTLTGKTITLEVEPSDTIENVKSKIQDKEGIPPDQQRLIFAGKQLEDGRTLSDYN

TRINITY_DN16432    57 IQKESTLHLVLRLRGGMQIFVKTLTGKTITLDVEPSDTIENVKSKIQDKEGIPPDQQSLI
BAB22630.1         61 IQKESTLHLVLRLRGGMQIFVKTLTGKTITLEVEPSDTIENVKAKIQDKEGIPPDQQRLI
AAA28997.1         61 IQKESTLHLVLRLRGGMQIFVKTLTGKTITLEVEPSDTIENVKAKIQDKEGIPPDQQRLI
PIK45544.1         61 IQKESTLHLVLRLRGGMQIFVKTLTGKTITLEVEPSDTIENVKSKIQDKEGIPPDQQRLI

TRINITY_DN16432   117 FAGKQLEDGRTLSDYNIQKESTLHLVLRLRGGMQIFVKTLTGKTITLDVEPSDTIENVKT
BAB22630.1        121 FAGKQLEDGRTLSDYNIQKESTLHLVLRLRGGMQIFVKTLTGKTITLEVEPSDTIENVKA
AAA28997.1        121 FAGKQLEDGRTLSDYNIQKESTLHLVLRLRGGMQIFVKTLTGKTITLEVEPSDTIENVKA
PIK45544.1        121 FAGKQLEDGRTLSDYNIQKESTLHLVLRLRGGMQIFVKTLTGKTITLEVEPSDTIENVKS

TRINITY_DN16432   177 KNQD-----------------------------------------------
BAB22630.1        181 KIQDKEGIPPDQQRLIFAGKQLEDGRTLSDYNIQKESTLHLVLRLRGGY--
AAA28997.1        181 KIQDKEGIPPDQQRLIFAGKQLEDGRTLSDYNIQKESTLHLVLRLRGGIQA
PIK45544.1        181 KIQDKEGIPQTSSV--------------SS---------SLVNN-------
```

D

```
TRINITY_DN52789     1 MIQKESTLHLVLRLRGGMQFFVKTLTGKTITLEVEPSDTIENLKYKIQDKKGIPQDQNRL
AAH19850.1          1 MQIFVKTLTGKTITLEVEPSDTIENVKAKIQDKEGIPPDQQRLIFAGKQLEDGRTLSDYN
NP_001284937.1      1 MQIFVKTLTGKTITLEVEPSDTIENVKAKIQDKEGIPPDQQRLIFAGKQLEDGRTLSDYN
PIK45544.1          1 MQIFVKTLTGKTITLEVEPSDTIENVKSKIQDKEGIPPDQQRLIFAGKQLEDGRTLSDYN

TRINITY_DN52789    61 IFDGKQLEDGRTLSVYMIQKESTLHLVLRLRGGMQFFVKTLTGKTITLEVEPSDTIENLK
AAH19850.1         61 IQKESTLHLVLRLRGGMQIFVKTLTGKTITLEVEPSDTIENVKAKIQDKEGIPPDQHRLI
NP_001284937.1     61 IQKESTLHLVLRLRGGMQIFVKTLTGKTITLEVEPSDTIENVKAKIQDKEGIPPDQQRLI
PIK45544.1         61 IQKESTLHLVLRLRGGMQIFVKTLTGKTITLEVEPSDTIENVKSKIQDKEGIPPDQQRLI

TRINITY_DN52789   121 YKIQDKKGIPQDQNRLIFDGKQLEDGRTLSDYNIQYDSTLDLVVPVGGCMQMWV------
AAH19850.1        121 FAGKQLEDGRTLSDYNIQKESTLHLVLRLRGGMQIFVKTLTGKTITLEVEPSDTIENVKA
NP_001284937.1    121 FAGKQLEDGRTLSDYNIQKESTLHLVLRLRGGMQIFVKTLTGKTITLEVEPSDTIENVKA
PIK45544.1        121 FAGKQLEDGRTLSDYNIQKESTLHLVLRLRGGMQIFVKTLTGKTITLEVEPSDTIENVKS

TRINITY_DN52789       ------------------------------------------------------------
AAH19850.1        181 KIQDKEGIPPDQQRLIFAGKQLEDGRTLSDYNIQKESTLHLVLRLRGGMQIFVKTLTGKT
NP_001284937.1    181 KIQDKEGIPPDQQRLIFAGKQLEDGRTLSDYNIQKESTLHLVLRLRGGMQIFVKTLTGKT
PIK45544.1        181 KIQDKEGIPQTSSVSSSLVNN---------------------------------------

TRINITY_DN52789       ------------------------------------------------------------
AAH19850.1        241 ITLEVEPSDTIENVKAKIQDKEGIPPDQQRLIFAGKQLEDGRTLSDYNIQKESTLHLVLR
NP_001284937.1    241 ITLEVEPSDTIENVKAKIQDKEGIPPDQQRLIFAGKQLEDGRTLSDYNIQKESTLHLVLR
PIK45544.1            ------------------------------------------------------------

TRINITY_DN52789       ------------------------------------------------------------
AAH19850.1        301 LRGGY-------------------------------------------------------
NP_001284937.1    301 LRGGMQIFVKTLTGKTITLEVEPSDTIENVKAKIQDKEGIPPDQQRLIFAGKQLEDGRTL
PIK45544.1            ------------------------------------------------------------

TRINITY_DN52789       ------------------------------------------------------------
AAH19850.1            ------------------------------------------------------------
NP_001284937.1    361 SDYNIQKESTLHLVLRLRGGMQIFVKTLTGKTITLEVEPSDTIENVKAKIQDKEGIPPDQ
PIK45544.1            ------------------------------------------------------------

TRINITY_DN52789       ------------------------------------------------------------
AAH19850.1            ------------------------------------------------------------
NP_001284937.1    421 QRLIFAGKQLEDGRTLSDYNIQKESTLHLVLRLRGGMQIFVKTLTGKTITLEVEPSDTIE
PIK45544.1            ------------------------------------------------------------

TRINITY_DN52789       ------------------------------------------------------
AAH19850.1            ------------------------------------------------------
NP_001284937.1    481 NVKAKIQDKEGIPPDQQRLIFAGKQLEDGRTLSDYNIQKESTLHLVLRLRGGAI
PIK45544.1            ------------------------------------------------------
```

Figure S1: (**A-D**) Alignment of each of the four ubiquitin *H. glaberrima* transcripts with their respective closest matches from the NCBI non-redundant database.
